# Dopaminergic Neurotoxicants Cause Biphasic Inhibition of Purinergic Calcium Signaling in Astrocytes

**DOI:** 10.1371/journal.pone.0110996

**Published:** 2014-11-03

**Authors:** Karin M. Streifel, Albert L. Gonzales, Briana De Miranda, Rola Mouneimne, Scott Earley, Ronald Tjalkens

**Affiliations:** 1 Center for Environmental Medicine, Colorado State University, Fort Collins, Colorado, United States of America; 2 Vascular Physiology Research Group, Department of Biomedical Sciences, Colorado State University, Fort Collins, Colorado, United States of America; 3 Department of Veterinary Integrative Biosciences, Texas A & M University, College Station, Texas, United States of America; Temple University School of Medicine, United States of America

## Abstract

Dopaminergic nuclei in the basal ganglia are highly sensitive to damage from oxidative stress, inflammation, and environmental neurotoxins. Disruption of adenosine triphosphate (ATP)-dependent calcium (Ca^2+^) transients in astrocytes may represent an important target of such stressors that contributes to neuronal injury by disrupting critical Ca^2+^-dependent trophic functions. We therefore postulated that plasma membrane cation channels might be a common site of inhibition by structurally distinct cationic neurotoxicants that could modulate ATP-induced Ca^2+^ signals in astrocytes. To test this, we examined the capacity of two dopaminergic neurotoxicants to alter ATP-dependent Ca^2+^ waves and transients in primary murine striatal astrocytes: MPP^+^, the active metabolite of 1-methyl 4-phenyl-1,2,3,6-tetrahydropyridine (MPTP), and 6-hydroxydopamine (6-OHDA). Both compounds acutely decreased ATP-induced Ca^2+^ transients and waves in astrocytes and blocked OAG-induced Ca^2+^ influx at micromolar concentrations, suggesting the transient receptor potential channel, TRPC3, as an acute target. MPP^+^ inhibited 1-oleoyl-2-acetyl-sn-glycerol (OAG)-induced Ca^2+^ transients similarly to the TRPC3 antagonist, pyrazole-3, whereas 6-OHDA only partly suppressed OAG-induced transients. RNAi directed against TRPC3 inhibited the ATP-induced transient as well as entry of extracellular Ca^2+^, which was augmented by MPP^+^. Whole-cell patch clamp experiments in primary astrocytes and TRPC3-overexpressing cells demonstrated that acute application of MPP^+^ completely blocked OAG-induced TRPC3 currents, whereas 6-OHDA only partially inhibited OAG currents. These findings indicate that MPP^+^ and 6-OHDA inhibit ATP-induced Ca^2+^ signals in astrocytes in part by interfering with purinergic receptor mediated activation of TRPC3, suggesting a novel pathway in glia that could contribute to neurotoxic injury.

## Introduction

Calcium (Ca^2+^) signaling modulates diverse physiological processes in the nervous system including exocytosis of neurotransmitters, neuronal long-term potentiation [1], [2], the propagation of intercellular Ca^2+^ waves in astrocytes that regulate metabolic and trophic support to neurons, and local regulation of cerebral blood flow [3], [4]. Adverse effects resulting from dysregulation of Ca^2+^ signaling in neurons during oxidative and inflammatory conditions has been extensively described [5] but the pathophysiological effect of alterations in Ca^2+^ signaling in astrocytes is less well understood. However, disruptions in astrocyte Ca^2+^ signaling could have serious implications for neuronal function and survival during states of neurological injury and disease. Transient increases in intracellular Ca^2+^ are essential for astrocytic regulation of neuronal excitability in both the healthy and diseased brain [6] and could therefore be an important target of both endogenous and exogenous neurotoxicants. Astrocytes tonically protect against excitotoxic neuronal injury not only by removing excess synaptic glutamate but also by dynamically inhibiting glutamatergic synapses through release of ATP that is degraded to adenosine and suppresses pre-synaptic currents via the A1 adenosine receptor [4]. Because astrocytic regulation of excitatory synapses requires Ca^2+^-dependent release of ATP, deprecations in Ca^2+^ signaling in astrocytes could predispose regional populations of neurons to excitotoxic injury. Ca^2+^ signaling in astrocytes could also be dysregulated during neuroinflammation, as indicated by recent studies which demonstrated that ATP release from activated microglia evokes P2Y purinergic receptor-dependent release of neuroactive compounds from astrocytes, including ATP, D-serine and glutamate [7]. Because activation of glia is a common pathological feature of multiple neurodegenerative conditions, alterations in astrocyte Ca^2+^ signaling could contribute to neuronal dysfunction following exposure to oxidative or neurotoxic insults or during conditions of chronic neuroinflammation.

Extracellular ATP activates purinergic receptors (P2 receptors) on astrocytes [8] that cause rapid increases in intracellular Ca^2+^. P2 receptors are divided into two subfamilies: ionotropic (P2X) receptors and metabotropic (P2Y) receptors. P2Y receptors are activated by low physiological ATP concentrations (<10 µM), whereas P2X receptors are activated by high concentrations of ATP that are released during pathological events such as ischemia and oxidative stress [9]. P2Y receptors are G-protein-coupled receptors (GPCR) that activate phospholipase-C (PLC)-dependent generation of inositol 1,4,5-triphosphate (IP_3_) that induces release of Ca^2+^ from the endoplasmic reticulum. Additionally, PLC activation generates diacylglycerol (DAG) that stimulates a more prolonged influx of extracellular Ca^2+^, resulting in a biphasic Ca^2+^ response that causes opening of receptor operated channels including TRPC3 [10]. However, how these Ca^2+^ influx pathways are affected by neurotoxic exposures is not well understood. This is important because sustained increases in [Ca^2+^]_i_ are required for exocytosis of ATP in astrocytes following purinergic stimulation [4].

A possible target of neurotoxic compounds that could decrease Ca^2+^ responses to purinergic signals is the transient receptor potential (TRP) channel, a family of selective and non-selective cation channels that are widely expressed in diverse cell types, including astrocytes [10]. Previous research in smooth muscle cells demonstrated that TRPC3 channels are associated with ATP-dependent Ca^2+^ signaling through activation of P2Y G_q_ GPCR receptors that activate the PLC-IP_3_-DAG pathway [11], [12]. DAG is a direct agonist for canonical TRPC3 channels [13], [14] that are abundant in the basal ganglia of both mouse [15] and human [16]. We therefore postulated that this Ca^2+^-permeable, non-selective cation channel is associated with vulnerability to the dopaminergic neurotoxicants, MPP^+^, the active metabolite of 1-methyl 4-phenyl 1,2,3,6-tetrahydropyridine (MPTP), and 6-hydroxydopamine (6-OHDA), an endogenous dopamine oxidation product that is generated at higher levels during aging. We propose a novel mechanism in astrocytes in which ATP-induced Ca^2+^ transients are acutely suppressed by neurotoxic cationic molecules through inhibition of purinergic signaling and through blockade of TRPC3 channel function, thereby altering the global amplitude of the intracellular Ca^2+^ response. To examine mechanisms by which these neurotoxicants could dysregulate ATP-induced Ca^2+^ signaling in astrocytes, we acutely exposed cultured striatal astrocytes to each compound and determined the effect on ATP- or OAG-induced Ca^2+^ transients using real-time fluorescence imaging, as well as the capacity of each compound to inhibit electrophysiologic Ca^2+^ currents in cells over-expressing TRPC3 and in primary astrocytes. The results of these studies indicated that acute application of each compound prior to stimulation with ATP or OAG decreased the amplitude of evoked transients, in addition to diminishing TRPC3-dependent electrophysiological currents induced by OAG. These data suggest that TRPC3 is a receptor-operated channel in astrocytes linked to the activation of metabotropic purinergic receptors and that selected cationic neurotoxicants suppress Ca^2+^ transients in astrocytes in part by inhibiting this pathway, implicating this channel as a target that may be relevant to neurotoxic injury from structurally diverse cationic molecules.

## Materials and Methods

### Materials and reagents

All chemical reagents were from Sigma-Aldrich (St. Louis, MO) unless otherwise stated. C57BL/6J mice were obtained from Jackson Laboratories (Bar Harbor, ME). Cell culture media and fluorescent dyes were obtained from Invitrogen (Carlsbad, CA).

### Cell culture

Primary astrocytes were isolated from 1–3 day old C57BL/6J mice from either cortex or striatum, as previously described [17], [18]. Brains were rapidly dissected, extracted and maintained in Minimal Essential Media (MEM) supplemented with Earle's Salts and L-glutamine (Hyclone, Logan, UT), with 10% Fetal Bovine Serum and 1% penicillin-streptomycin-neomycin (Invitrogen, Carlsbad, CA). Cells were grown at 37°C, 5% CO_2_ in a humidified incubator for approximately three weeks until reaching a mature phenotype. Confluent astrocytes were sub-cultured onto 4-well poly-D-lysine-coated chambered glass slides (Nalgene-Nunc, Thermo Fisher Scientific, Waltham, MA) and allowed to grow to approximately 75% confluence. In our laboratory, cultures consistently yield>98% astrocytes as determined by immunofluorescence staining for glial fibrillary acidic protein [19].

### Ethics Statement

All procedures involving animals were conducted under a protocol approved by the Animal Care and Use Committee at Colorado State University (IACUC Protocol No. 12-3646A). According to the guidelines of the National Institutes of Health, every effort was made to minimize pain and discomfort and all terminal procedures for isolation of primary cells were conducted under deep isofluorane anesthesia.

### Calcium Imaging

Astrocytes were grown to approximately 75% confluence on 4-well chamber slides and incubated with Fluo-4 AM (2 µM) (Molecular Probes, Life Technologies, Carlsbad, CA) for 15 minutes at 37°C prior to imaging. Cells were imaged in media (MEM, without phenol red or sodium bicarbonate) supplemented with 10 mM HEPES (pH 7.4) at 25°C. Groups of approximately 15–30 contiguous cells per field of view were identified for imaging. Cells were stimulated with ATP (1 µM) to selectively activate metabotropic purinergic receptors, rather than ionotropic receptors [9] or with 1-oleoyl-2-acetyl-sn-glycerol (OAG; 100 µM), a direct TRPC channel agonist [13]. Compounds (MPP^+^, 6-OHDA, or 3,4-dihydroxyphenylacetic acid/DOPAC) were added 30 seconds prior to each agonist. Images of Fluo-4 fluorescence were collected every 500 milliseconds for 120 seconds with camera binning set at 4×4 pixels and an exposure time of 20 milliseconds. Images were collected on a Zeiss Axiovert 200 M microscope equipped with a Hammatsu ORCA-ER cooled charge-coupled device camera. Fluorescent intensity was expressed relative to the baseline image (F/F_0_), where F_0_ is the fluorescence level prior to stimulation. Datasets were analyzed using Slidebook software (v5.0; Intelligent Imaging Innovations, Inc., Denver, CO).

### Mechanical stimulation of calcium waves

For Ca^2+^ wave propagation studies, astrocytes were sub-cultured onto poly-D-lysine coated 30 mm round glass coverslips and placed in a flow chamber (POCmini, Carl Zeiss, New York, NY). After a 10 second collection of baseline Fluo-4 intracellular Ca^2+^ intensity, wave studies were mechanically-induced with a 5 µm diameter glass micropipet using a micromanipulator, as described previously [19]. Either MPP^+^, 6-OHDA or DOPAC (100 µM) were added 30 seconds prior to stimulation and images were acquired every 500 milliseconds for 60 seconds. Fluorescent intensity was expressed relative to the baseline image (F/F_0_) in all cells within the wave activation site. Wave amplitude and distance were determined using Slidebook software (v5.0; Intelligent Imaging Innovations, Inc., Denver, CO).

### Imaging ATP-induced calcium transients

Astrocytic Ca^2+^ transients were determined by real-time fluorescence imaging following stimulation with ATP (1 µM) or OAG (100 µM) in the presence of the phospholipase C (PLC) inhibitor, U73122 (10 µM), or its inactive analog, U73433 (10 µM). After a 10 min preincubation period with the inhibitor or analog, the cells were stimulated with 1 µM ATP or 100 µM OAG and images of Fluo-4 fluorescence collected every 500 milliseconds for 60 seconds. Fluorescent intensity was expressed relative to the baseline image (F/F_0_) in all cells. Pyrazole 3 (Pyr3, 10 µM), a selective extracellular inhibitor of TRPC3, was incubated with astrocytes for 30 minutes prior to imaging at 37°C. Following incubation with Pyr3, cells were stimulated with ATP or OAG as described. For experiments without extracellular Ca^2+^, Ca^2+^-free imaging medium supplemented with 2 mM EGTA (pH 7.4) was used in replacement of imaging medium, with all other experimental conditions held constant. Luciferase measurements were made using a 96-well luminescence plate reader (Molecular Dyanmics, Sunnyale, CA).

### Electrophysiology

HEK 293 cells were cultured in Dulbecco's 1× high glucose modified Eagle's medium supplemented with 10% fetal bovine serum and 0.5% penicillin-streptomycin (Gibco, Grand Island, NY). Cells were incubated at 37°C with 6% CO_2_ and sub-cultured when confluent using 0.25% trypsin-EDTA (Gibco). HEK 293 cells were transfected by electroporation with a plasmid that encodes TRPC3 channels tagged with yellow fluorescent protein (TRPC3-YFP)[20]. Transfected cells were plated on 12-mm glass coverslips and allowed to adhere for ∼2–3 h at 37°C. Transfected cells expressing TRPC3-YFP were identified using fluorescent microscopy. For patch clamp recordings from isolated astrocytes, confluent cells were sub-cultured onto glass coverslips in Minimal Essential Media and allowed to adhere for 2–3 hours at 37°C in 5% CO_2_ humidified incubator. Currents were recorded using an AxoPatch 200B amplifier equipped with an Axon CV 203BU headstage (Molecular Devices). Recording electrodes (1–3 MΩ) were pulled, polished, and coated with wax to reduce capacitance. GΩ seals were obtained in Mg-PSS. Currents were filtered at 1 kHz, digitized at 40 kHz, and stored for subsequent analysis. Clampex and Clampfit versions 10.2 (Molecular Devices) were used for data acquisition and analysis, respectively. All patch-clamp experiments were performed at 25°C. The extracellular bathing solution contained (mM) 140 NaCl, 5 CsCl, 0.1 MgCl_2_, 0.05 CaCl_2_, 10 Glucose, 10 HEPES (pH 7.4). Pipette solution contained (mM) 130 CsOH, 110 aspartic acid, 15 CsCl, 1 MgCl_2_, 3.6 CaCl_2_, 10 EGTA, 10 HEPES (pH 7.4). The calculated reversal potentials for monovalent and divalent cations are 0 mV and −24 mV, respectively. Reversal potentials for monovalent anions are 1.6 mV for these solutions. Cells were initially voltage clamped at 0 mV, and voltage ramps from −100 mV to +100 mV were applied every 4 seconds. After baseline currents were recorded, OAG (100 µM) or ATP (1 µM) was administered to activate TRPC3 currents. When the evoked current reached a steady state MPP^+^ (100 µM), 6-hydroxydopamine, 6-OHDA (100 µM), or Pyr3 (10 µM) were administered to examine the effect of these compounds on OAG- or ATP-induced TRPC3 currents.

### Expression of Purinergic receptors and TRP channels

Total RNA from cultured astrocytes were prepared using the RNeasy Mini Kit (Qiagen, Valencia, CA) with on-column DNase digestion. RNA was quantified using a Nanodrop system (Thermo Scientific, Wilmington, DE), with the average yield of RNA from 75–100 ng/μL. Whole brain total RNA was prepared with the Qiagen RNeasy Mini Kit and used for a positive control. cDNA was synthesized using reverse transcriptase (BioRad, Hercules, California) and reverse transcriptase-PCR was conducted. PCR products were visualized with a 1.5% agarose gel with ethidium bromide staining on a BioRad Chemidoc XRS imager. Primers used are listed in Table S1.

### TRPC3 knockdown

Primary striatal astrocytes (70% confluency) were transfected for 72 hrs with 25–50 pmol of siRNA oligonucleotides (Ambion/Life Technologies, Grand Island, NY) using the Transit-X2 reagent (Mirus Bio, Madison, WI) per the manufacturer's protocol. Knockdown was confirmed by reverse transcriptase PCR.

### Statistical analysis

Differences between three or more means with one independent variable was performed using one-way analysis of variance (ANOVA) followed by the Tukey's multiple comparison post hoc test using Prism software (v4.0c, Graphpad Software, Inc., San Diego, CA). Two-component variant analysis was performed using two-way ANOVA and Sidak's multiple comparison test. Comparisons of two groups were analyzed using the Student's t-test. Results are expressed as the mean ± SEM from a minimum of 3 independent studies and for all experiments, p<0.05 was considered significant.

## Results

### Acute exposure to toxicants inhibits Ca^2+^ transients in striatal astrocytes

**Figure 1:**
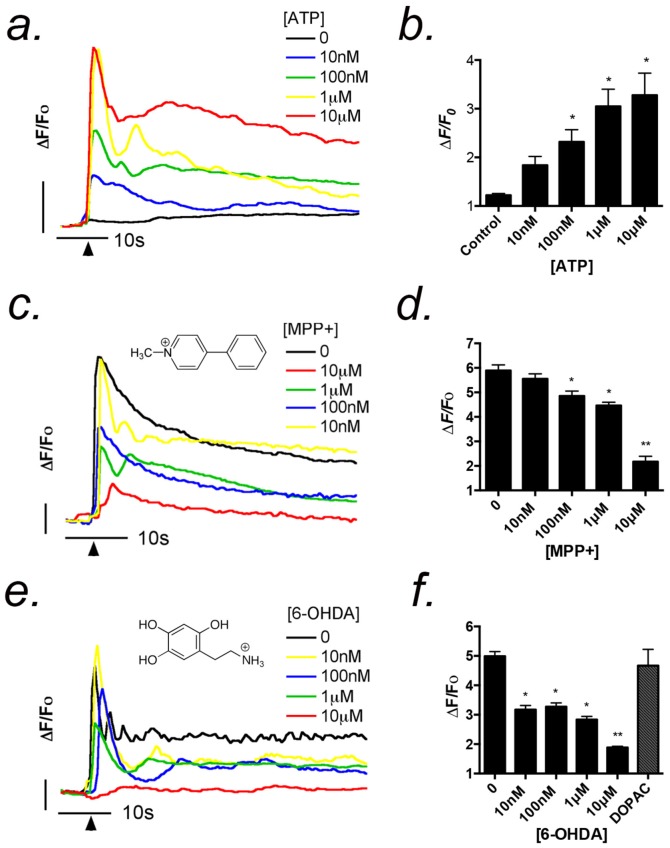
Acute exposure to dopaminergic neurotoxicants inhibits Ca^2+^ transients in striatal astrocytes. (a,b) Application of 10 nM–10 µM ATP resulted in Ca^2+^ transients in striatal astrocytes that persisted for greater than 60 sec. (c,d) Addition of MPP^+^ to the imaging media at increasing concentrations acutely suppressed ATP-induced Ca^2+^ transients. Representative traces of astrocytic responses to 1 µM ATP (black arrowhead indicates time of ATP addition) in the presence of MPP^+^ (10 nM–10 µM) indicate dose-dependent suppression of intracellular Ca^2+^ transients. (e,f) Application of 6-OHDA to the imaging media acutely suppressed ATP-induced Ca^2+^ transients. Representative traces of the response to 1 µM ATP in the presence of 6-OHDA (10 nM–10 µM) indicate dose-dependent suppression of intracellular Ca^2+^ transients, where as the dopamine metabolite, DOPAC (10 µM), did not affect the amplitude of Ca^2+^ transients. (a,c,e) Vertical bars denote 1 relative fluorescent unit; horizontal bars denote 10 sec. n = 3 independent experiments; data collected from 50–60 cells group from 2–3 microscopic fields; *p<0.05 relative to control, ** *p*<0.01 relative to control and treated groups.

The propagation of intercellular Ca^2+^ waves between astrocytes depends heavily upon release of ATP by SNARE proteins (soluble N-ethylmaleimide-sensitive-factor attachment protein receptor) and connexin 43 hemi-channels, which is stimulated by a transient rise in the intracellular Ca^2+^ concentration [4], [8]. The capacity of low concentrations of dopaminergic neurotoxicants to inhibit ATP-induced Ca^2+^ transients was investigated in Figure 1. Concentrations of ATP from 10 nM–10 µM selectively activate P2Y purinergic receptors and caused a dose-dependent rise in intracellular Ca^2+^ in primary striatal astrocytes, as determined by real-time fluorescence microscopy (Figure 1a,b). The initial peak of Ca^2+^ release from intracellular stores was followed by a sustained increase in Ca^2+^ associated with entry from the extracellular space. The acute effects of 1-methyl-4-phenylpyridinium ion (MPP^+^) and 6-hydroxydopamine (6-OHDA) on ATP-induced Ca^2+^ transients were determined by adding increasing concentrations of each neurotoxicant to the medium 30 sec prior to stimulation with ATP. This was done to test the direct effect of each cationic neurotoxicant on Ca^2+^ response independent of longer term alterations such as changes in gene expression or channel translocation. Increasing concentrations of MPP^+^ from 0.01 to 10 µM caused a concentration-dependent decrease in the maximum amplitude of ATP-induced Ca^2+^ transients in striatal astrocytes (Figure 1c,d). Acute application of 6-OHDA, an endogenous oxidation of product of dopamine that is positively charged at physiological pH, caused a similar dose-dependent decrease in intracellular Ca^2+^ in response to ATP (Figure 1e,f). Inhibition of ATP-induced intracellular Ca^2+^ transients by 6-OHDA was selective, because the dopamine metabolite, 3,4-dihydroxyphenylacetic acid (DOPAC; 10 µM), had no inhibitory effect on ATP-induced transients (Figure 1e,f). Expression of all P2 receptors was determined in primary astrocytes (Table S1) by reverse transcriptase PCR (rtPCR). Both cortical and striatal astrocytes expressed all P2X receptor subtypes and all P2Y receptor subtypes, with the exception of P2Y4 and P2Y13, which were not detected in cortical astrocytes (Table S1a,b). Expression of TRPC channels was similarly determined by rtPCR and indicated that subtypes 1–4 of TRPC channels were expressed in primary cortical astrocytes (Table S1c).

### Acute exposure to MPP^+^ and 6-OHDA attenuates Ca^2+^ wave propagation in primary astrocytes

**Figure 2:**
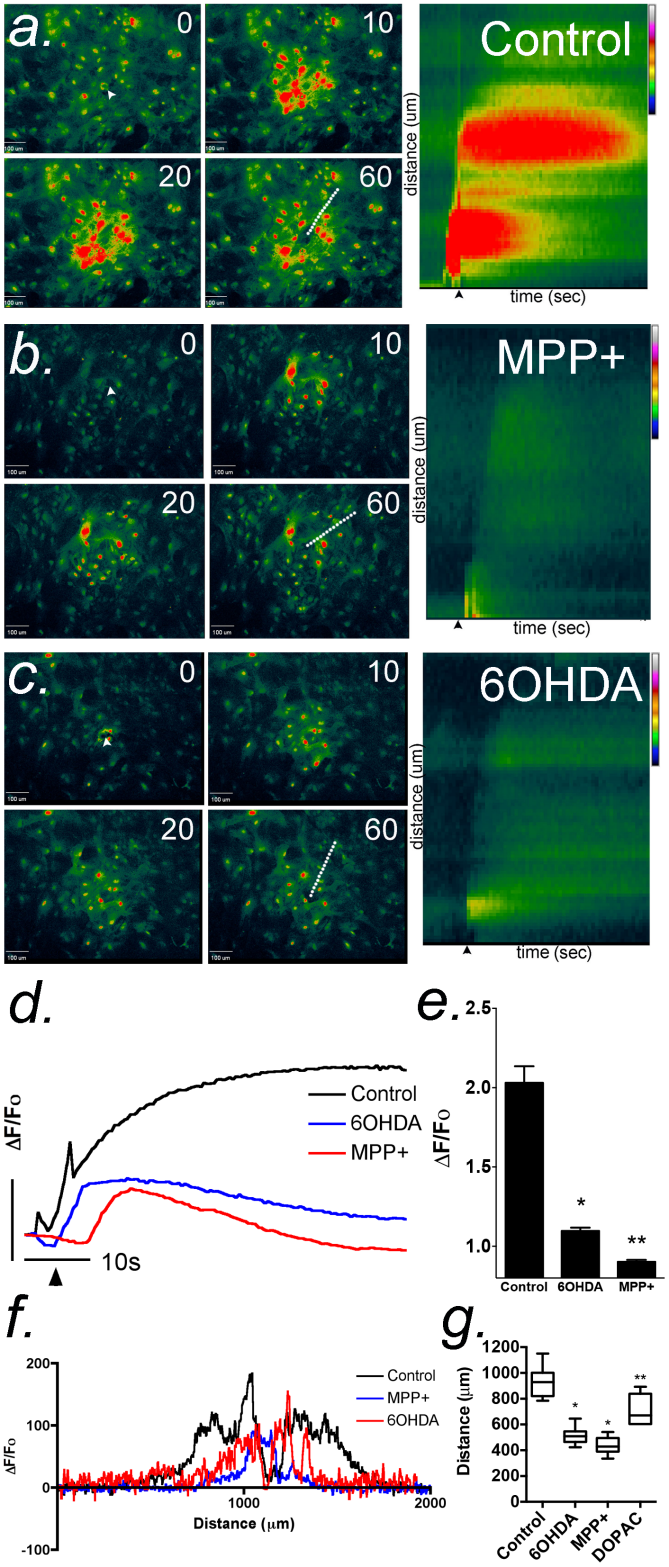
Acute exposure to dopaminergic neurotoxicants attenuates the propagation of intercellular Ca^2+^ waves in primary astrocytes. Calcium waves were initiated in confluent cultures of striatal astrocytes loaded with Fluo-4 AM (2 µM) using a glass micropipette to stimulate a single astrocyte in the center of the field (white arrow). Images were collected every 500 msec for 60 sec. (a) Control wave and kymograph images were generated from the fluorescence intensity of Fluo-4 along a representative line drawn from the point of stimulation to the terminus of the Ca^2+^ wave across across the field of astrocytes (a, dotted line). Black arrows denote the point of stimulation. (b) The extent and intensity of Ca^2+^ waves is sharply diminished by acute application of 100 µM MPP+. (c) Similarly to MPP+, 100 µM 6-OHDA diminished the extent and intensity of Ca^2+^ waves with acute application. (d,e) The mean intensity of intracellular Ca^2+^ responses in the cells acutely exposed to 100 µM MPP+ and 6-OHDA are significantly decreased compared to control. The vertical bar in (d) denotes 1 relative fluorescent unit; the horizontal bar denotes 10 sec. (f,g) Representative traces of the total distance of the wave front in control and acutely-treated cells. Quantitative analysis of Ca^2+^ wave propagation indicates that 100 µM MPP+ and 6-OHDA decreases the distance traveled in striatal astrocytes by>50% relative to control, whereas 100 µM DOPAC does not abolish wave distance like MPP+ and 6-OHDA although significantly decreased from control. n = 3 waves analyzed per group for each experiment over 3–4 independent experiments in separate cultures of striatal astrocytes; *p<0.05 relative to control, ** *p*<0.01 relative to control and treated groups.

Based upon our previous data that the divalent metal, Mn^2+^, acutely inhibits mechanically-induced Ca^2+^ waves in cultured striatal astrocytes [21], we postulated that MPP^+^ and 6-OHDA might similarly diminish Ca^2+^ wave activity by targeting membrane cation channels. The data in Figure 2 demonstrate that mechanical stimulation of a single astrocyte in the center of a confluent field using a 0.5 µm glass micropipet initiated Ca^2+^ waves that propagated outward from the stimulated cell for approximately 500 µm, reaching maximal extent within 60 sec. The amplitude of the wavefront over time is indicated by the kymograph images in Figure 2, representing the fluorescence intensity along the line shown in the image panel at 60 sec following stimulation. Acute application of either MPP^+^ or 6-OHDA (100 µM each) 30 sec prior to stimulation of Ca^2+^ waves resulted in a dramatic decrease in the amplitude of the wavefront throughout the field of astrocytes. Quantification of the maximum intensity of the intracellular Ca^2+^ transient at the wavefront in each group is shown in Figure 2d,e. 100 µM MPP^+^ was more effective than 6-OHDA at suppressing mechanically-induced Ca^2+^ waves in striatal astrocytes, although both compounds resulted in greater than 50% inhibition of the maximum amplitude of the wavefront. 100 µM MPP^+^ and 6-OHDA were equally effective in diminishing the distance of propagation of Ca^2+^ waves, whereas acute administration of 100 µM DOPAC resulted in only a slight decrease in the extent of wave propagation (Figure 2f,g).

### Metabotropic receptors mediate ATP-induced Ca^2+^ transients from intracellular and extracellular sources

**Figure 3:**
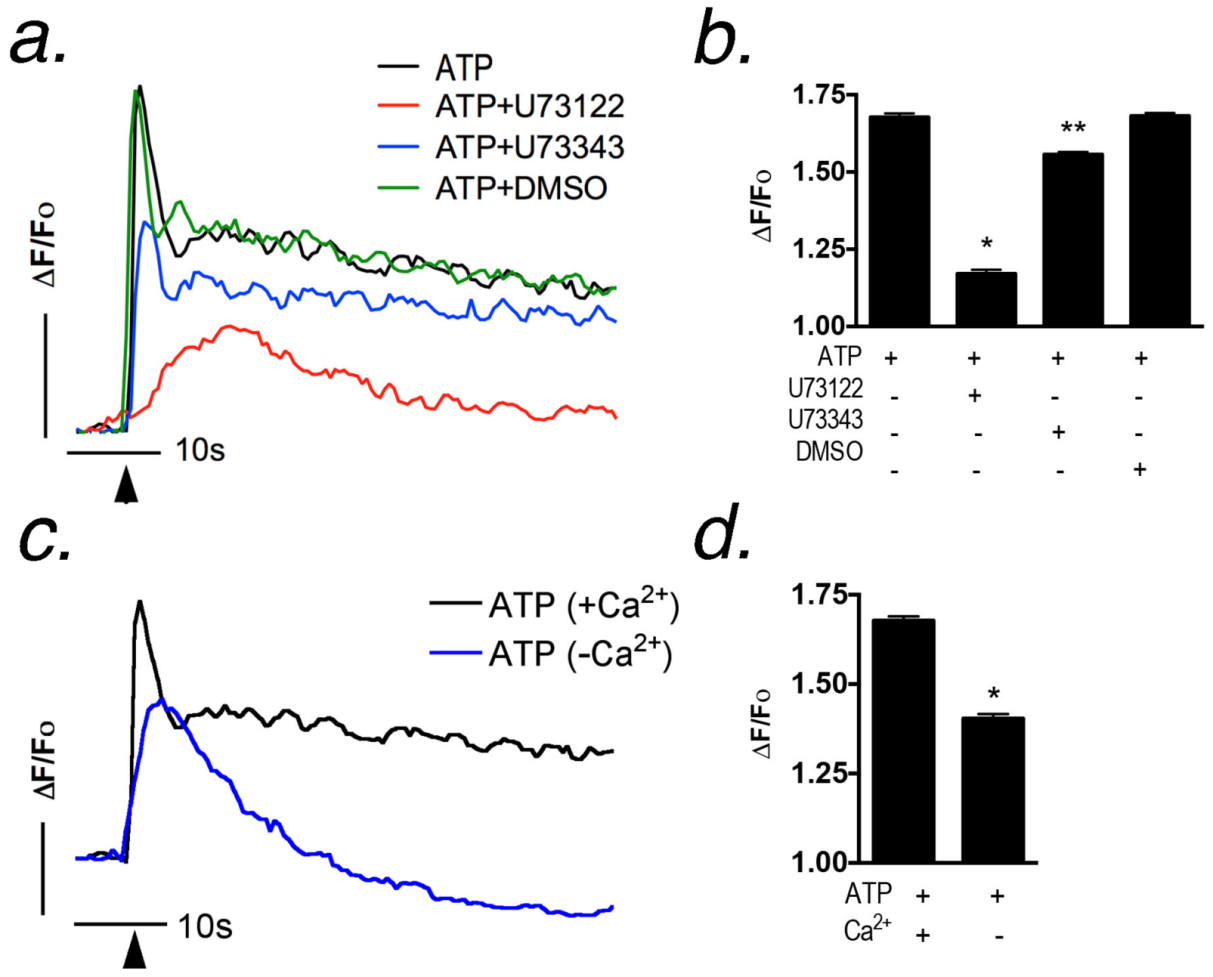
ATP stimulates both metabotropic receptors and entry of extracellular Ca^2+^ in striatal astrocytes. (a,b) Ca^2+^ transients were determined in astrocytes by real-time fluorescence imaging following stimulation with 1 µM ATP in the presence of the phospholipase C inhibitor, U73122 (10 µM), its inactive analog, U73343 (10 µM), or vehicle control (DMSO). Black arrows denote the time of addition of 1 µM ATP. (c–d) Removal of extracellular Ca^2+^ inhibited both the peak amplitude and sustained phase of the Ca^2+^ transient. The vertical bar (a,c) denotes 1 relative fluorescent unit; the horizontal bar denotes 10 sec. n = 500–700 cells analyzed per experiment over 2–3 independent experiments in separate cultures of striatal astrocytes; *p<0.05 relative to control, ** *p*<0.01 relative to control and treated groups.

In order to identify the site of inhibition of dopaminergic toxicants on ATP-induced Ca^2+^ signaling responses in astrocytes, we first characterized the relative contribution of intracellular and extracellular Ca^2+^ in shaping the amplitude and extent of the ATP-induced transient. Addition of 1 µM ATP caused a rapid increase in intracellular Ca^2+^ in striatal astrocytes (Figure 3a) that was decreased by the PLC inhibitor U73122 (10 µM). Inhibition of PLC suppressed the initial peak of Ca^2+^ release from intracellular stores following addition of ATP, as well as the resultant receptor-operated influx of Ca^2+^ influx from the extracellular space (Figure 3a,b). An inactive analog of U73122, U73343 (10 µM), had only a very slight effect on the amplitude of the ATP-induced Ca^2+^ transient, whereas the vehicle control (DMSO) had no inhibitory effect. Stimulation with 1 µM ATP in Ca^2+^-free imaging medium reduced the peak amplitude of the Ca^2+^ transient but abolished the sustained phase of Ca^2+^ entry from the extracellular space (Figure 3c,d). These data indicate that extracellular Ca^2+^ is an important component of the intracellular transient in astrocytes following stimulation of PLC-dependent purinergic receptors with low concentrations of ATP.

### OAG elicits Ca^2+^ transients in astrocytes independently of intracellular Ca^2+^ release

**Figure 4:**
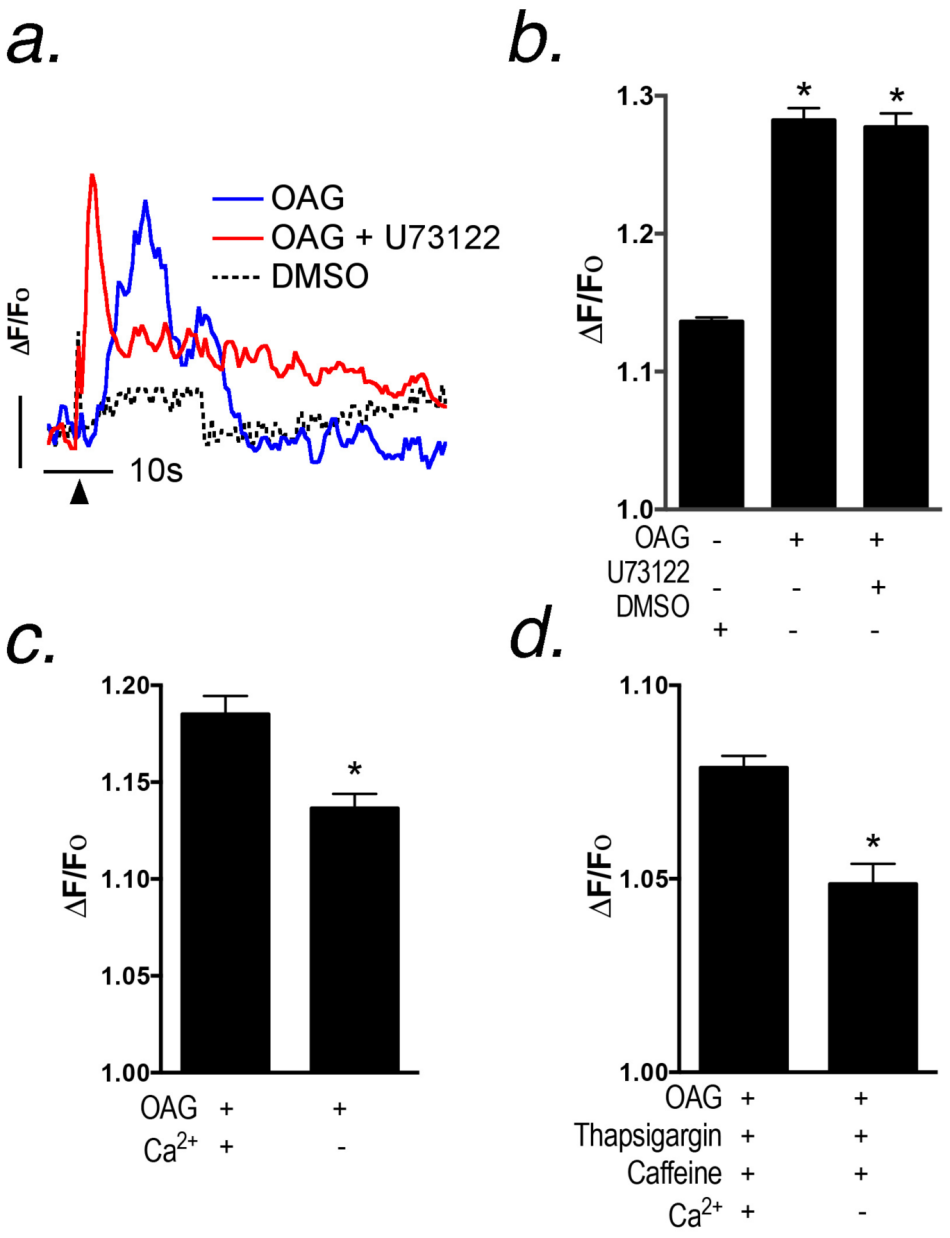
OAG activates Ca^2+^ transients in astrocytes independently of the phospholipase C - IP_3_ pathway that require extracellular Ca^2+^ but do not require Ca^2+^ release from intracellular stores. (a,b) Ca^2+^ transients were stimulated with 100 µM OAG in the presence or absence of the PLC inhibitor, U73122 (10 µM), or DMSO vehicle control. Quantification of peak amplitudes from (a) indicates that 10 µM U73122 had no inhibitory effect on OAG-induced Ca^2+^ transients, suggesting that OAG causes Ca^2+^ entry separate from the PLC-IP_3_ pathway. The vertical bar in (a) denotes 1 relative fluorescent unit; the horizontal bar denotes 10 sec. Black arrows denote the point of stimulation by 100 µM OAG. (c) Ca^2+^ transients were initiated with 100 µM OAG in the presence or absence of extracellular Ca^2+^, demonstrating a significant inhibition in OAG response with the removal of extracellular Ca^2+^. (d) Intracellular Ca^2+^ stores were depleted by successive additions of 10 µM thapsigargin and 5 mM caffeine prior to OAG-induced Ca^2+^ transients in order to determine the role of intracellular and extracellular Ca^2+^. Results indicate that prior depletion of intracellular Ca^2+^ stores had no effect on OAG-induced Ca^2+^ transients yet depletion of extracellular Ca^2+^ inhibited the amplitude of OAG-induced transients similar to (c). n = 500–700 cells for each experiment over 2–3 independent experiments in separate cultures of striatal astrocytes; *p<0.05.

Activation of P2Y receptors by ATP generates both IP_3_ that induces release of Ca^2+^ from intracellular stores, as well as diacylglycerol (DAG) that can trigger influx of extracellular Ca^2+^ through plasma membrane TRP channels [12], [22]. To determine the contribution of DAG-dependent Ca^2+^ entry in astrocytes to the overall ATP-induced transient, we stimulated astrocytes with the DAG analog, 1-oleoyl-2-acetyl-sn-glycerol (OAG), a membrane permeable analog of DAG, that directly activates TRPC 3, 6 and 7 [13] (Figure 4). TRPC3, but not TRPC6 or 7, is expressed in astrocytes cultured by our methods (Figure 2c), consistent with other reports indicating that TRPC3 is abundant in glial cells [10]. Addition of OAG (100 µM) to primary astrocytes resulted in an increase in intracellular Ca^2+^ that was not prevented by pretreatment with U73122 (10 µM; Figure 4a,b). Removal of Ca^2+^ from the extracellular medium prevented OAG-induced increases in intracellular Ca^2+^ (Figure 4c). However, OAG stimulation was still able to elicit a Ca^2+^ transient following depletion of intracellular Ca^2+^ stores by pretreatment with thapsigargin (10 µM) and caffeine (5 mM; Figure 4d). Thus, ATP-dependent activation of P2Y receptors in astrocytes results in influx of extracellular Ca^2+^ that contributes to the overall amplitude and shape of the intracellular transient.

### GPCR and TRPC channel Ca^2+^ responses are decreased by inhibition of TRPC3 and by dopaminergic neurotoxicants

**Figure 5:**
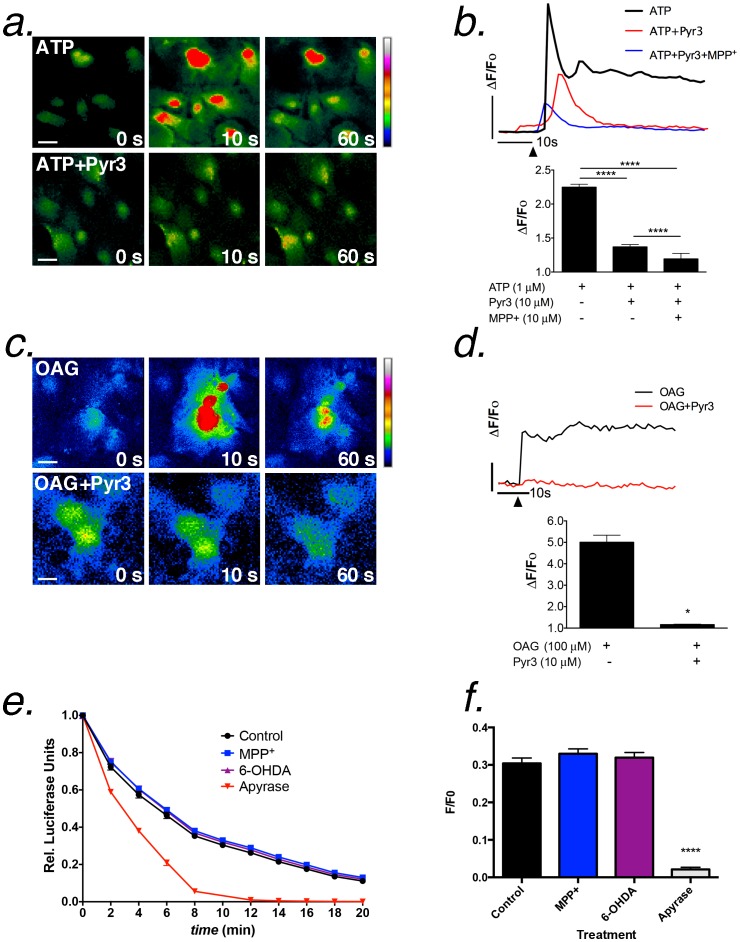
Inhibition of TRPC3 decreases ATP- and OAG-induced Ca^2+^ transients in striatal astrocytes. (a,b) Changes in Fluo-4 fluorescence in striatal astrocytes l following stimulation with 1 µM ATP in presence or absence of the TRPC3 inhibitor, pyrazole-3 (Pyr3; 10 µM). Prior treatment with Pyr3 for 30 minutes at 37°C decreased the Ca^2+^ response to ATP, whereas only a modest additive effect was observed with Pyr3 in the presence of 10 µM MPP^+^. (c,d) Addition of 100 µM OAG induced a rapid increase in Ca^2+^ that was abolished by pre-incubation with Pyr3. Vertical bars in (b and c) denote 1 relative fluorescent unit; the horizontal bars denote 10 sec. Black arrows indicate the time of stimulation with ATP or OAG. Representative images are shown at the start of imaging (time = 0 sec) and at the application of agonist (time = 10 sec) and again at time = 60 sec. Scale bar  =  10 microns. (e,f) Luciferase (40 ng/ml) was incubated with luciferin and ATP at 22°C for 20 min in the presence of MPP^+^ (10 µM) 6-OHDA (10 µM) or apyrase (5 U/ml). For imaging studies, n = 500–700 cells for each experiment over 2-3 independent experiments in separate cultures of striatal astrocytes; for luciferase experiments, n = 4 independent replicates. *p<0.05 relative to control, ** *p*<0.01 relative to control and treated groups, **** *p*<0.0001 compared to control, MPP^+^ and 6-OHDA.

Because DAG/OAG directly regulates entry of extracellular Ca^2+^ following stimulation of astrocytes with physiological concentrations of ATP, we posited that inhibition of TRP channels would inhibit both ATP- and OAG-induced Ca^2+^ transients. This hypothesis was tested by the studies in Figure 5, which used the TRPC3 inhibitor, Pyr3, to prevent entry of extracellular Ca^2+^ [23]. These data demonstrate that intracellular Ca^2+^ transients in astrocytes induced by stimulation with ATP (1 µM) were suppressed, although not completely abolished, by pretreatment (60 sec) with Pyr3 (10 µM; Figure 5a,b). The sustained phase of Ca^2+^ entry was inhibited to a greater extent by Pyr3 than the initial intracellular transient. Combined exposure to Pyr3 (10 µM) and MPP^+^ (10 µM) further reduced ATP-induced Ca^2+^ transients, although the initial response to ATP was still present. Pyr3 completely blocked the intracellular response to OAG (100 µM). (Figure 5c,d). To determine whether MPP^+^ and 6-OHDA could interfere purinergic receptor activation by directly modulating ATP as a biochemical cofactor, we conducted *in vitro* assays in which purified luciferase was incubated with ATP and luciferin in the presence of each neurotoxin or apyrase, which degrades ATP to adenosine (Figure 5e,f). Neither MPP^+^ or 6-OHDA showed any inhibitory effect on ATP-dependent luciferase activity by 10 min (Figure 5f, *p*<0.0001), whereas ATP degradation by apyrase rapidly diminished the luminescence signal.

**Figure 6:**
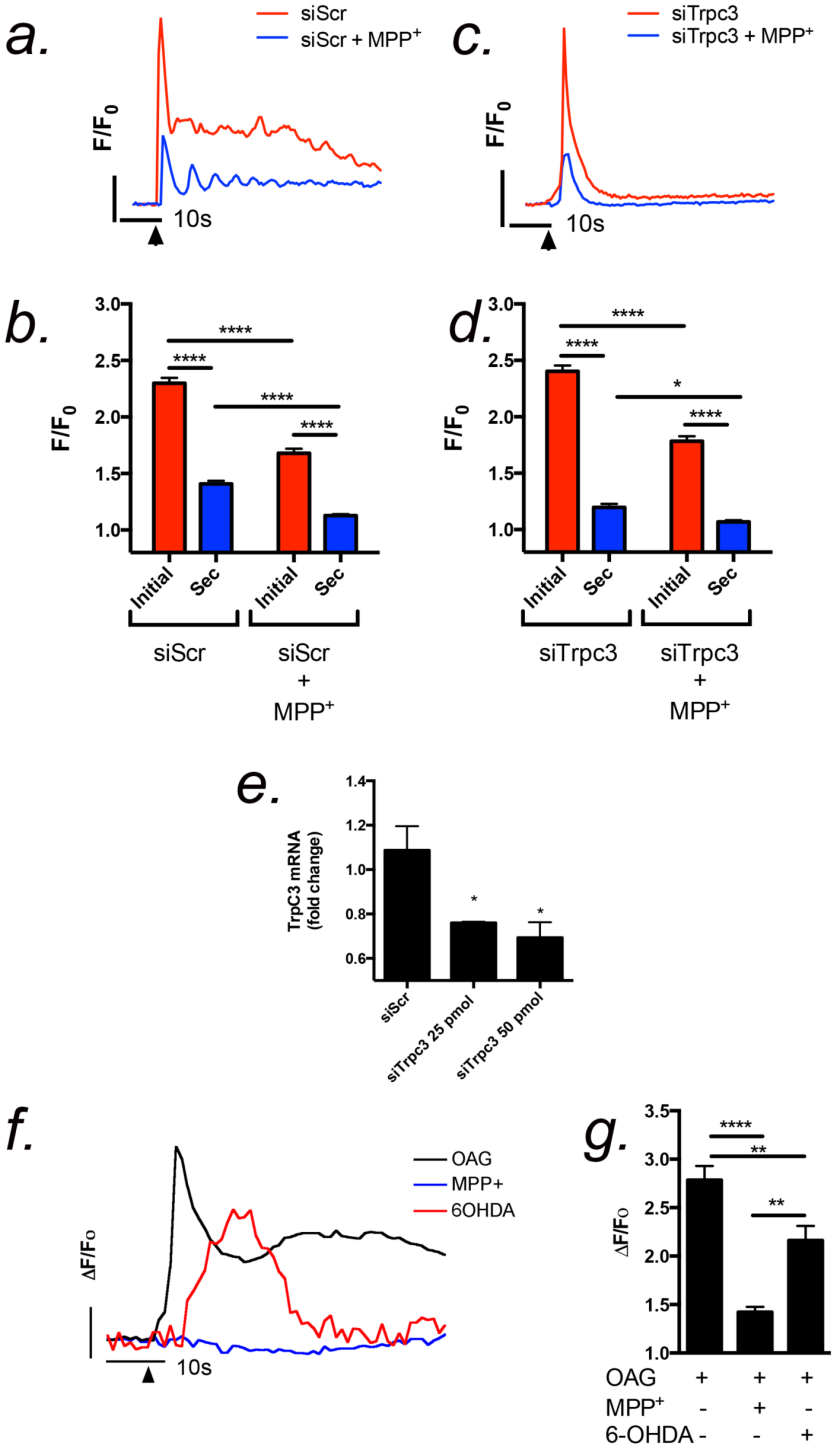
MPP^+^ and 6-OHDA acutely inhibit OAG-induced Ca^2+^ transients in striatal astrocytes. (a-d) Knockdown of TRPC3 in striatal astrocytes using siRNA decreases extracellular Ca^2+^ entry (secondary peak height) but not the initial ATP-induced transient (initial peak height). MPP^+^ only slightly enhances the inhibitory effect of TRPC3 siRNA on ATP-induced extracellular Ca^2+^ entry. (e) Expression of TRPC3 mRNA following 72 hrs knockdown with siRNA oligonucleotides. Black arrows indicate the time of stimulation with 1 µM ATP. (f) Representative traces demonstrating changes in OAG-induced Ca^2+^ influx following the addition of cationic neurotoxins. The vertical bar denotes 1.0 relative fluorescent unit; the horizontal bar denotes 10 sec. Black arrows indicate the time of stimulation by 100 µM OAG. (g) Quantitative analysis of Fluo-4 fluorescence demonstrates that OAG-induced activation of TRPC3 channels is decreased in the presence of MPP^+^ and 6-OHDA (10 µM each). n = 200–300 cells for each experiment over 2–3 independent experiments in separate cultures of striatal astrocytes; *p <0.05, **p <0.01, ****p <0.0001.

Knockdown of TRPC3 in primary astrocytes using siRNA oligonucleotides resulted in attenuation of extracellular Ca^2+^ entry but did not affect IP_3_-dependent transients following stimulation with ATP (Figure 6). Cells transfected with scrambled control oligonucleotides responded robustly to ATP with a large initial Ca^2+^ transient followed by a delayed plateau phase of extracellular Ca^2+^ entry (Figure 6a,b). The initial Ca^2+^ peak was diminished by MPP^+^ but not as severely as the secondary plateau phase of extracellular Ca^2+^ entry. Knockdown of TRPC3 (>50%; Figure 6e) resulted in a significant attenuation of the secondary extracellular Ca^2+^ entry but had minimal effect on the IP_3_-dependent transient (Figure 6c,d). MPP^+^ partley inhibited the initial ATP-induced Ca^2+^ transient following TRPC RNAi and further reduced the residual plateau phase of Ca^2+^ below that caused by TRPC3 RNAi alone (Figure 6c,d). To determine whether MPP^+^ and 6-OHDA had a similar effect on OAG-dependent Ca^2+^ signaling in astrocytes, Ca^2+^ responses to exogenously applied OAG (100 µM) were measured after 30 sec pre-incubation with each compound (Figure 6f,g), similar to pretreatments conducted with Pyr3 in Figure 5. OAG-induced Ca^2+^ transients were significantly attenuated by MPP^+^ (10 µM) compared to control (Figure 6f). 6-OHDA (10 µM), in contrast, diminished but did not completely suppress the OAG-induced Ca^2+^ transient, although the time to peak amplitude was delayed and the sustained phase of the Ca^2+^ transient was decreased (Figure 6g). These results suggest that TRPC3 modulates ATP-dependent Ca^2+^ signals in astrocytes associated largely with the sustained phase of Ca^2+^ entry although TRPC3 inhibitors also modestly attenuate the IP_3_-dependent release from intracellular stores.

### MPP^+^ inhibits TRPC3-like currents in astrocytes

**Figure 7:**
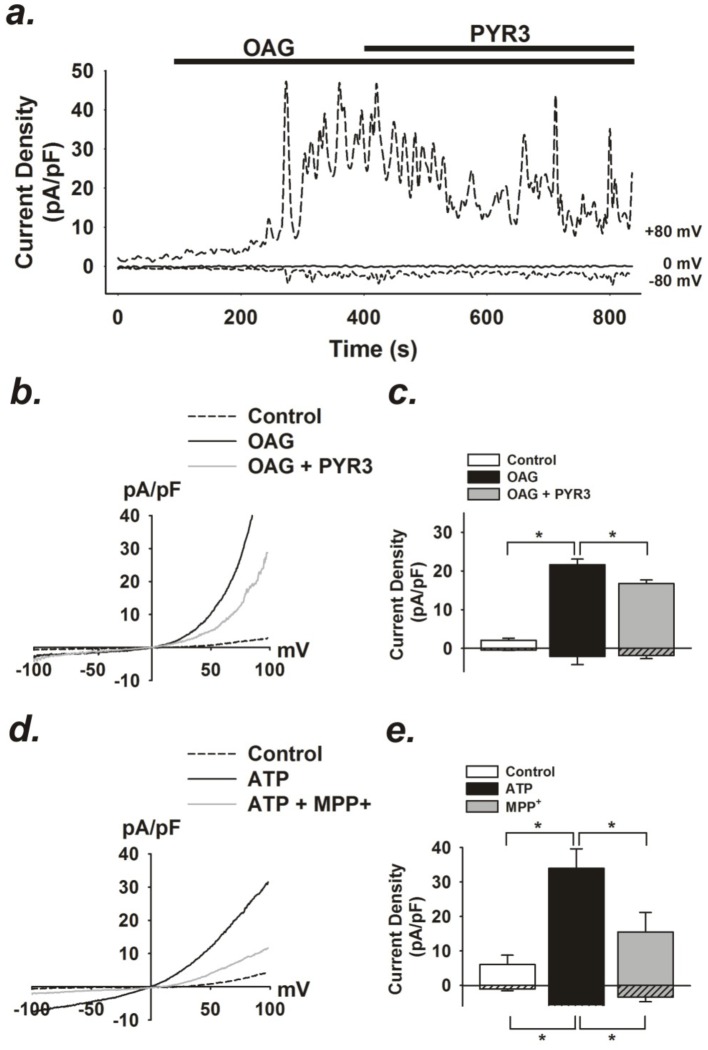
OAG- and ATP-induced currents in striatal astrocytes. (a) Representative time course of OAG (100 µM) -induced whole-cell currents at +80, 0, and −80 mV and in response to TRPC3 inhibitor, pyrazole-3 (Pyr3, 10 µM) in striatal astrocytes. (b) Current (I) vs. voltage (V) relationship of baseline and OAG-induced currents in absence and presence of Pyr3. (c) Summary data of average current density at +80 and −80 mV before and following OAG stimulation and after administration of Pyr3. n  =  5. (*p <0.05). (c) Current (I) vs. voltage (V) relationship of baseline and ATP (1 µM) -induced currents in striatal astrocytes in absence and presence of MPP^+^ (100 µM). (d) Summary data of average current density at +80 and −80 mV before and following ATP (1 µM) stimulation and after administration of MPP^+^ (100 µM). n  =  3. (*p <0.05).

To determine whether MPP^+^-dependent inhibition of Ca^2+^ signaling correlated to a loss of TRPC3 current, we performed whole-cell patch clamp electrophysiology experiments to isolate native TRPC3 current in primary striatal astrocytes. Large transient outward currents were evoked following the administration of OAG (100 µM), and were blocked with the TRPC3 inhibitor, Pyr3 (10 µM, Figure 7a–c), supporting the presence of native TRPC3 currents in isolated astrocytes. Due to a possible global effect of OAG on counteracting K^+^ currents, we tested whether local generation of DAG through P2Y receptors could specifically activate TRPC3-like currents in astrocytes. Consistent with Ca^2+^ imaging experiments, administration of extracellular ATP generated large inward (and outward) currents that were blocked by MPP^+^ (Figure 7d–e).

### TRPC3 currents are differently affected by MPP^+^ and 6-OHDA

**Figure 8:**
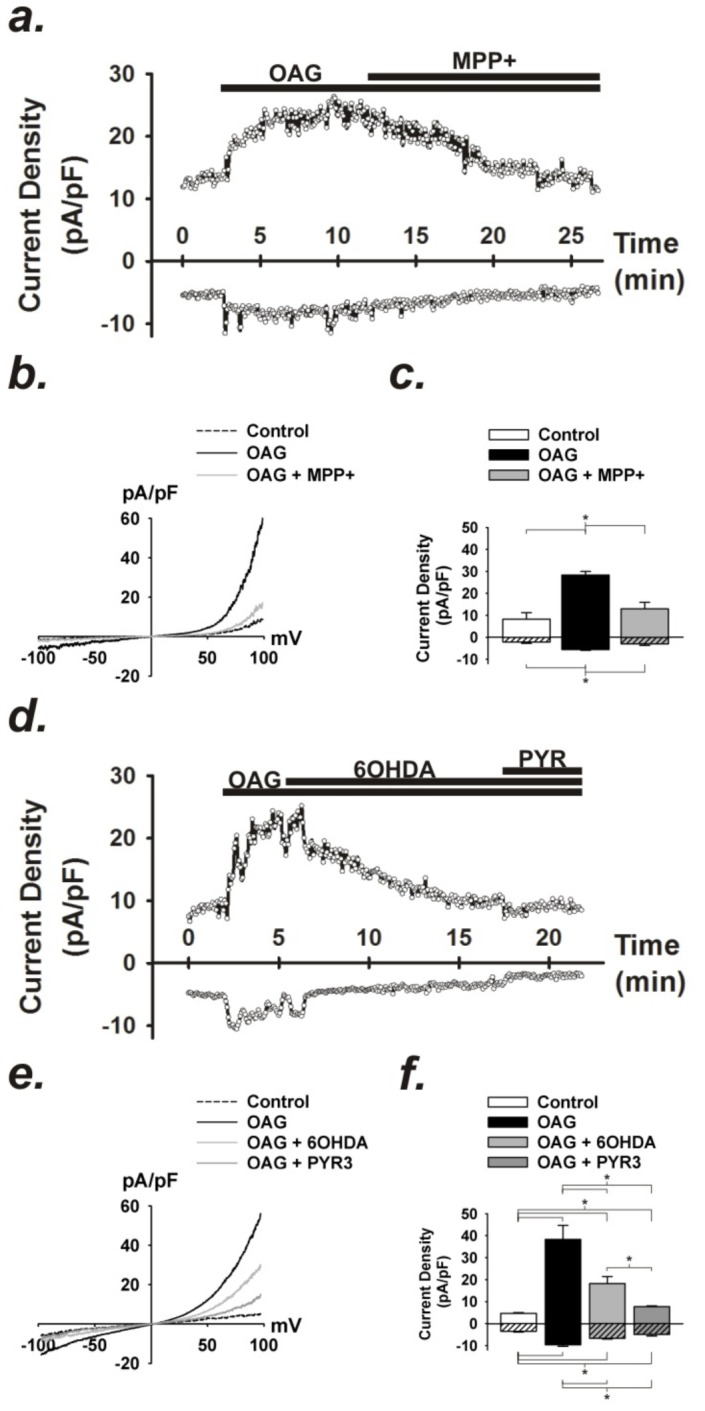
OAG-induced currents are blocked by MPP^+^ and 6-OHDA in cells overexpressing TRPC3. (a) Representative time course of OAG (100 µM) -induced whole-cell currents at +80 and −80 mV and in response to of MPP^+^ (100 µM) in human embryonic kidney (HEK) cells overexpressing TRPC3-YFP. (b) Current (I) vs. voltage (V) relationship of baseline and OAG-induced currents in absence and presence of MPP^+^. (c) Summary data of average current density at +80 and −80 mV before and following OAG stimulation and after administration of MPP+. n  =  4. (*p <0.05). (d) Representative time course of OAG (100 µM) -induced whole-cell currents at +80 and −80 mV and in response to of 6-OHDA (100 µM) and PyR3 (10 µM). (e) Current (I) vs. voltage (V) relationship of baseline and OAG-induced currents recorded in absence and presence of 6-OHDA or PyR3. (f) Summary data of average current density at +80 and −80 mV before and following OAG stimulation and after administration of 6-OHDA or Pyr3. n  =  4. (*p <0.05).

To directly measure the effects of MPP^+^ and 6-OHDA on TRPC3 channels, recombinant TRPC3 was expressed in HEK 293 cells and channel currents were examined by electrophysiology using the whole-cell patch clamp technique (Figure 8). Using the overexpression of TRPC3 in HEK293 cells, we can have a high signal-to-noise ratio to better isolate the currents, directly activate the channel with OAG independent of K^+^ channels, and can examine direct pharmacological effects of MPP^+^ and 6-OHDA on TRPC3 channels. Control currents from unstimulated HEK cells were initially small but large currents were evoked following the administration of the membrane permeable DAG analog, OAG (100 µM) (Figure 7a,c). OAG-dependent currents in TRPC3-expressing HEK cells were blocked by the TRPC3 inhibitor, Pyr3 (10 µM; Figure 8d–f), indicating that the measured currents originated from TRPC3 channel activity. OAG-induced TRPC3 currents were also strongly inhibited by administration of 100 µM MPP^+^ (Figure 7b), whereas 100 µM 6-OHDA only partially inhibited OAG-induced currents, less than either MPP^+^ or Pyr3 (Figure 7c,d). These results further implicate TRPC3 as a target of MPP^+^-mediated inhibition.

## Discussion

Calcium (Ca^2+^) signaling in astrocytes is essential for modulating diverse physiological processes in the CNS, including synaptic transmission, neurovascular coupling and metabolic activity in glial cells [4]. Altered purinergic signaling in astrocytes has been associated with neurodegenerative diseases that affect the basal ganglia, including Parkinson's disease [24], but mechanisms underlying pathophysiological changes in Ca^2+^ signaling during disease states and neurotoxic stress are not well understood. We therefore examined whether selected toxicants of the dopaminergic system could alter ATP-dependent puringergic Ca^2+^ signaling in astrocytes in part by acutely inhibiting TRPC3 channels, which could provide insight into novel glial-specific signaling mechanisms underlying the sensitivity of dopaminergic nuclei to oxidative and neurotoxic injury. Although these toxins are structurally distinct and represent endogenous (6-OHDA) and exogenous (MPP^+^) insults, they may share a common mechanism of action in their capacity to acutely disrupt astrocytic Ca^2+^ responses to purinergic stimuli that are critical for maintaining neuronal homeostatic and trophic functions.

Cultured astrocytes express P2X and P2Y purinergic receptors, as well as numerous canonical TRPC channels, including TRPC3 (Figure S1). Levels of ATP released during CNS injury can reach 1 mM and preferentially activate P2X ionotropic receptors in astrocytes, whereas physiological ATP levels up to 10 µM activate P2Y metabotropic receptors [9]. Striatal astrocytes responded to ATP with robust intracellular Ca^2+^ transients that were inhibited by both MPP^+^ and 6-OHDA at low micromolar concentrations (Figure 1), characterized by a decrease in the peak amplitude of the initial transient and a more pronounced suppression of the sustained phase of the signal associated with entry of Ca^2+^ from the extracellular space. This suggests a direct effect on the activity of channels associated with capacitive or receptor-operated Ca^2+^ entry following acute exposure to these neurotoxins, although there may also be a direct effect on GPCR activity of P2Y receptors. Additionally, it is possible that either the dopamine transporter or the organic cation transporter (OCT) could influence certain long term intracellular effects of MPP^+^ or 6-OHDA. Although these channels are expressed in astrocytes [25], the rapid timescale of inhibition and the lack of bidirectional transport by OCT makes these channels less likely to acute targets of MPP^+^ and 6-OHDA [26]. 6-OHDA causes ROS formation that can inhibit PLC activity in neuronal cells after 24–72 hrs of treatment [27] but the short exposure periods used in the present studies and the lesser effect of 6-OHDA compared to MPP^+^ likely precludes ROS formation as a primary mechanism. Furthermore, acute addition of DOPAC had no effect on ATP-induced Ca^2+^ transients (Figure 1e,f), demonstrating that only cationic 6-OHDA was inhibitory and not the anionic carboxylic acid metabolite of dopamine. Because other GPCR's have been linked to receptor-operated Ca^2+^ channels [12], we postulated that the observed acute inhibitory effect of these toxicants could involve direct alteration of such channels in addition to any direct effects on purinergic receptors.

Acute application of MPP^+^ or 6-OHDA to confluent cultures of primary astrocytes also inhibited intercellular Ca^2+^ waves (Figure 2). This demonstrates the inhibitory effects of these compounds in a system that better models *in vivo* cell-cell communication, because Ca^2+^ waves are highly dependent on purinergic receptor signaling. P2Y receptors are critical to propagation of intercellular Ca^2+^ waves in astrocytes [28] and both MPP^+^ and 6-OHDA suppressed the intensity and propagation distance of mechanically induced waves. This indicates that these compounds not only inhibit ATP-mediated intracellular Ca^2+^ transients following exogenous stimulation of P2Y receptors but also during paracrine release of ATP from astrocytes during intercellular calcium wave propagation. It was reported in astrocyte cultures prepared from optic nerve that ATP concentrations at the site of mechanical stimulation with a micropipet can reach 78 µM but that concentrations of ATP released only 100 micrometers distant from the stimulated cell are approximately 7 µM [29], which would therefore selectively activate P2Y in the majority of cells in the field. We found that the intensity of the Ca^2+^ wave 75 micrometers from site of activation was decreased by both MPP^+^ and 6-OHDA, relative to control waves (kymograph images in Figure 2a–c), suggesting that P2Y-dependent Ca^2+^ responses induced by cell-cell communication between astrocytes are acutely inhibited by these compounds, similar to the effect observed on ATP-induced transients. Intracellular Ca^2+^ transients induced by direct application of ATP were inhibited by MPP^+^ and 6-OHDA at lower concentrations than those required to suppress intercellular waves, likely due to the involvement of transmitters in addition to ATP that mediate Ca^2+^ transients during wave propagation. Astrocytic Ca^2+^ waves are known to involve the release of transmitters in addition to ATP, such as glutamate, that augment wave activity [4] and possibly mobilize distinct Ca^2+^ stores from those activated by ATP, potentially increasing the concentration of cationic neurotoxin required to inhibit wave activity.

To identify possible sites of inhibition of MPP^+^ and 6-OHDA on ATP-induced Ca^2+^ transients in striatal astrocytes, we first determined the relative contribution of intracellular vs. extracellular Ca^2+^ to the overall amplitude of the transient following stimulation with ATP. The PLC inhibitor, U73122, significantly decreased the maximum amplitude of ATP-induced intracellular Ca^2+^ transients following stimulation with 1 µM ATP (Figure 3a,b), indicating a requirement for GPCR-dependent activation of the PLC-IP_3_ pathway in ATP-induced purinergic signaling. However, there was still a small residual ATP-induced transient in the presence of U73122, consistent with previous studies demonstrating that blockade of PLC signaling in astrocytes with either 2-APB (2-aminoethyl diphenylborinate) or PMA (phorbol myristate acetate) was unable to completely abolish ATP-induced Ca^2+^ responses [10]. Removal of extracellular Ca^2+^ only partially decreased the amplitude of the initial ATP-induced transient but completely abolished the sustained phase of the Ca^2+^ response associated with entry of extracellular Ca^2+^ (Figure 3c,d), indicating that Ca^2+^ from the extracellular space is important in shaping the amplitude and duration of the intracellular transient following stimulation with ATP. To further examine the potential site of inhibition of Ca^2+^ influx, we used the membrane-permeable analog of DAG, 1-oleoyl-2-acetyl-sn-glycerol (OAG), which directly activates TRPC3 channels [13]. Previous studies indicated that TRPC3 is expressed in astrocytes [30] and we also identified mRNA for TRPC3 and several other TRP family members in striatal astrocytes (Figure S1, Table S1), suggesting that this DAG/OAG-sensitive channel could be an important target of MPP^+^ and 6-OHDA. Expression of DAG-sensitive TRPC6/7 was not detected in primary striatal astrocytes, further supporting TRPC3 as the likely site of inhibition of extracellular Ca^2+^ entry.

OAG directly induced Ca^2+^ influx in striatal astrocytes independent of PLC, because U73122 failed to inhibit OAG-dependent increases in intracellular Ca^2+^ (Figure 4a,b). Additionally, depletion of extracellular Ca^2+^ abolished OAG-induced increases in intracellular Ca^2+^, whereas prior depletion of intracellular Ca^2+^ with caffeine and thapsigargin had no effect (Figure 4c,d), indicating that OAG directly induces Ca^2+^ influx through a plasma membrane cation channel in astrocytes. We postulated that this channel was TRPC3, because the TRPC3 antagonist, Pyr3, decreased both ATP- and OAG-induced Ca^2+^ transients in striatal astrocytes (Figure 5), although inhibition of OAG-induced transients was greater than ATP-induced transients. Combined treatment with Pyr3 and MPP^+^ further diminished the IP_3_-dependent initial transient induced by ATP compared to either compound alone but both had a similar inhibitory effect on the plateau phase of Ca^2+^ entry, suggesting that blockade of TRPC3 may have a feedback inhibitory effect Ca^2+^-induced Ca^2+^ release from intracellular stores. The effects of MPP^+^ and 6-OHDA on ATP-induced transients was not due to direct binding and/or interference with the activity of ATP as a co-factor, because neither compound inhibited luciferase activity in biochemical assays (Figure 5e,f). In contrast, degradation of ATP with apyrase resulted in rapid loss of luminescence.

**Figure 9:**
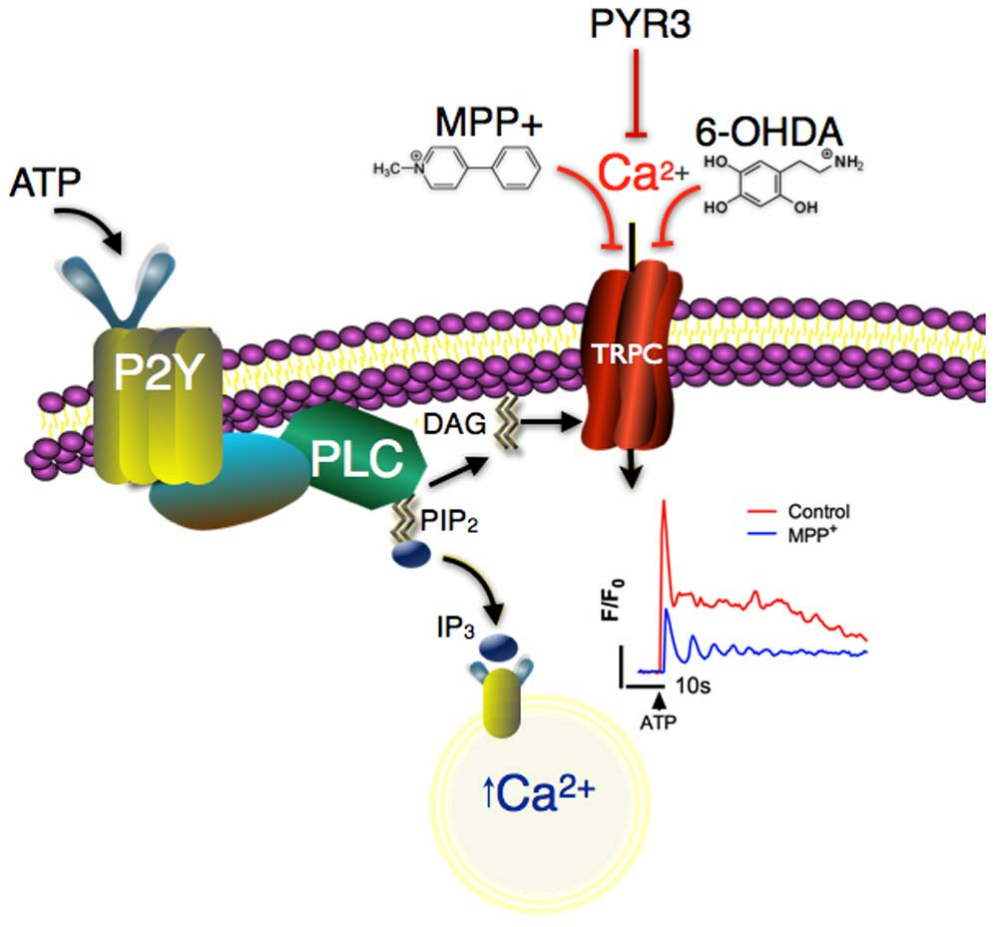
Proposed mechanism by which cationic neurotoxicants disrupt ATP-induced calcium signaling in astrocytes. This scheme represents a novel pathophysiologic mechanism that calcium signaling in astrocytes is an important target of selected dopaminergic neurotoxicants. Adenosine triphosphate (ATP) at nanomolar to micromolar concentrations activates metabotropic purinergic receptors (P2Y), causing phospholipase C (PLC)-dependent release of inositol triphosphate (IP_3_) and diacylglycerol (DAG) from phosphatidylinositol 4,5-bisphosphate (PIP_2_). IP_3_ stimulates release of Ca^2+^ from the endoplasmic reticulum, represented by the initial peak in the trace (blue) and DAG activates TRPC channels, allowing influx of cations, including Ca^2+^, from the extracellular space, represented by the sustained phase of the trace (red). In the presence of MPP^+^ or 6-OHDA, Ca^2+^ influx is disrupted following stimulation with ATP or OAG, thereby diminishing the net movement of Ca^2+^ into the cell.

The extent of inhibition of the sustained phase of Ca^2+^ entry by Pyr3 during ATP-induced transients suggests that P2Y-dependent generation of IP_3_/DAG and subsequent activation of TRPC3 channels following stimulation with ATP is critical to maximal mobilization of Ca^2+^ in striatal astrocytes and dramatically influences the overall amplitude of the intracellular Ca^2+^ transient. RNAi knockdown of TRPC3 dramatically attenuated the sustained phase of Ca^2+^ entry following the initial IP_3_-mediated transient in primary astrocytes, supporting the importance of TRPC3 to sustained phase of Ca^2+^ entry (Figure 6a-d, *p*<0.0001). The addition of MPP^+^ to TRPC3 RNAi cells abolished the residual peak associated with entry of extracellular Ca^2+^, supporting that TRPC3 is an important target of MPP^+^ in addition to the observed direct effects on purinergic signaling. Accordingly, acute application of MPP^+^ attenuated OAG-induced Ca^2+^ transients in striatal astrocytes, whereas 6-OHDA was only partially suppressed OAG-induced transients compared to control (Figure 6). Combined with the electrophysiology data in Figures 8 and 9, these findings suggest that MPP^+^ is a higher affinity inhibitor of TRPC3 than 6-OHDA. These results are also consistent with previous findings from our laboratory that Pyr3 was more effective at inhibiting OAG- vs. ATP-induced Ca^2+^ entry in astrocytes, based upon Fura-2 quenching studies [21], consistent with the specificity of OAG for this channel subtype.

The direct effects of MPP^+^ and 6-OHDA on the activity of TRPC3 channels were determined by whole-cell patch clamp electrophysiology in primary striatal astrocytes (Figure 7). These studies determined that OAG evoked large transient outward currents that were partly blocked with Pyr3, suggesting the presence of this channel *in situ*. Interestingly, we were unable to record an inward current following the administration of OAG. This may be due to non-specific PKC-dependent activation of two-pore-domain K^+^ (K_2P_) channels [31], a major hyperpolarizing contributor to the passive conductance of mature astrocytes [32]. OAG-sensitive activation of K^+^ efflux via K_2P_ channels could oppose any potential Na^+^/Ca^2+^ influx via TRPC3 in native cells, yielding little or no net charge movement and could explain the lack of inward current at hyperpolarizing potentials in our patch clamp recordings. In contrast, ATP evoked large inward and outward currents in astrocytes that were suppressed by both Pyr3 and MPP^+^ (Figure 7), suggesting that stimulation of P2Y receptors and the PLC-dependent production of local DAG could specifically activate native TRPC3-like current in astrocytes that are disrupted by MPP^+^. In cells overexpressing TRPC3 (Figure 8), electrophysiology data indicated that both inward and outward TRPC3-dependent Ca^2+^ currents were blocked by MPP^+^ (Figure 8a,b), providing evidence for direct interaction with this TRPC channel subtype. TRPC3 currents were also partially decreased by 6-OHDA, relative to Pyr3, but to a lesser extent than MPP^+^, suggesting a weaker interaction with TRPC3 for this compound.

Ca^2+^ responses to ATP could also implicate intracellular targets including the ryanodine receptor (RyR). This receptor is inhibited by toxicants such as polybrominated diphenyl ethers (PBDE's) and non-coplanar polychlorinated biphenyl compounds (PCB's), which alter Ca^2+^ homeostasis in neural cells [33], [34]. Although MPP^+^ is non-coplanar [35] and could therefore potentially react with RyR, the rapid kinetics of the inhibitory effect with MPP^+^ and 6-OHDA more strongly suggest that they function as channel blockers at the plasma membrane, as demonstrated in recent studies with the divalent metal, Mn^2+^, which reversibly inhibited OAG-induced Ca^2+^ transients in astrocytes by competitively decreasing Ca^2+^ influx through TRPC3 [21]. The electrophysiological data in the present studies also suggests that MPP^+^ and, to a lesser extent 6-OHDA, acutely decreases ATP-dependent Ca^2+^ transients in astrocytes by partly by inhibiting the channel activity of TRPC3. These data do not preclude the possibility of longer term changes in Ca^2+^ signaling due to altered gene expression or translocation of TRPC3 [36], [37], but rather implicate TRPC3 as an important acute target of these cationic neurotoxicants that can negatively modulate Ca^2+^ responses of astrocytes to ATP.

Interestingly, TRPC3 is also expressed in neurons, where it is associated with postsynaptic excitatory currents at glutamatergic synapses. TRPC3 knockout mice display no alterations in mobilization of Ca^2+^ from intracellular stores but have deprecations in mGluR-mediated inward currents, indicating an important role for TRPC3 in stimulus-dependent Ca^2+^ entry [38]. Our data indicate that a similar phenomenon may be operative in astrocytes, where inhibition of TRPC3 by MPP^+^ or 6-OHDA decreases ATP-dependent Ca^2+^ transients partly by blocking entry of extracellular Ca^2+^, thereby suppressing downstream signaling responses to critical gliotransmitters such as ATP that are associated with Ca^2+^ wave activity and intercellular communication between astrocytes (Figure 9). It is interesting to speculate that MPP^+^ or 6-OHDA could also directly affect TRPC3 in neurons, causing a general suppression of postsynaptic excitatory activity, but addressing this question would require further investigation.

Collectively, these data indicate that ATP-induced Ca^2+^ signaling in striatal astrocytes is vulnerable to inhibition by structurally distinct cationic neurotoxicants through suppression of purinergic signaling and by inhibition of extracellular Ca^2+^ entry through TRPC3. The present study therefore presents a novel mechanism by which selected dopaminergic neurotoxins can acutely inhibit purinerigic Ca^2+^ signaling in astrocytes. The observed alterations in glial Ca^2+^ homeostasis could therefore have negative effects on neuronal function in states of injury and disease.

## Supporting Information

Figure S1 **Expression of P2X/Y and TRPC receptors in primary astrocytes.** (a) Expression of ionotropic P2X receptors in primary cortical and striatal astrocytes was determined by reverse transcriptase-PCR (rtPCR) and indicated that all P2X receptor subtypes are expressed in both cortical and striatal astrocytes. (b) Metabotropic P2Y receptors were broadly expressed in cortical astrocytes except P2Y4 and P2Y13, whereas striatal astrocytes expressed all P2Y receptors. (c) Expression of TRPC subfamily receptors was determined by rtPCR. TRPC receptors 1–4 were detected in primary astrocytes. Results are representative of 2 independent experiments in separate cultures of primary astrocytes.
(DOCX)

Table S1 **Expression of P2X/Y and TRPC receptors in primary astrocytes.** (a) Expression of ionotropic P2X receptors in primary cortical and striatal astrocytes was determined by reverse transcriptase-PCR (rtPCR) and indicated that all P2X receptor subtypes are expressed in both cortical and striatal astrocytes. (b) Metabotropic P2Y receptors were broadly expressed in cortical astrocytes except P2Y4 and P2Y13, whereas striatal astrocytes expressed all P2Y receptors. (c) Expression of TRPC subfamily receptors was determined by rtPCR. TRPC receptors 1–4 were detected in primary striatal astrocytes. Results are representative of 2 independent experiments in separate cultures of primary astrocytes.
(DOCX)
