# Supplementary material for: Dopaminergic Neurotoxicants Cause Biphasic Inhibition of Purinergic Calcium Signaling in Astrocytes
Source: PLoS One. 2014 Nov 3;9(11):e110996. doi: 10.1371/journal.pone.0110996 (PMC4217743; doi:10.1371/journal.pone.0110996)
Supplement: Table S1 — Expression of P2X/Y and TRPC receptors in primary astrocytes. (a) Expression of ionotropic P2X receptors in primary cortical and striatal astrocytes was determined by reverse transcriptase-PCR (rtPCR) and indicated that all P2X receptor subtypes are expressed in both cortical and striatal astrocytes. (b) Metabotropic P2Y receptors were broadly expressed in cortical astrocytes except P2Y4 and P2Y13, whereas striatal astrocytes expressed all P2Y receptors. (c) Expression of TRPC subfamily receptors was determined by rtPCR. TRPC receptors 1–4 were detected in primary striatal astrocytes. Results are representative of 2 independent experiments in separate cultures of primary astrocytes. (DOCX) [file pone.0110996.s002.docx]

**Supplemental Table 1. Expression of P2X/Y and TRPC receptors in primary astrocytes.** (a) Expression of ionotropic P2X receptors in primary cortical and striatal astrocytes was determined by reverse transcriptase-PCR (rtPCR) and indicated that all P2X receptor subtypes are expressed in both cortical and striatal astrocytes. (b) Metabotropic P2Y receptors were broadly expressed in cortical astrocytes except P2Y4 and P2Y13, whereas striatal astrocytes expressed all P2Y receptors. (c) Expression of TRPC subfamily receptors was determined by rtPCR. TRPC receptors 1-4 were detected in primary striatal astrocytes. Results are representative of 2 independent experiments in separate cultures of primary astrocytes.

a.

| Receptor | Cortical | Striatal | Primer 5’🡪3’ | Accession No | BP |
| --- | --- | --- | --- | --- | --- |
| P2X1 | + | + | ACACGTGTGAAGATCTTTGGCTGGT  ACAGCGGGTGTAGAATCTTGTGGT | NM008771 | 210 |
| P2X2 | + | + | TGGACAGGCAGGGAAATTCAGTCT  TGGACATGGTTACTGAAGAGCGCA | NM153400 | 566 |
| P2X3 | + | + | TATCCTTGGGCTGCAGAACCTTCA  TATTGCATTTCCCTGGGCTCTCCT | NM14526 | 523 |
| P2X4 | + | + | TCATCCTGGCTTACGTCATTGGGT  ACACCTTCCAGTCCCAATTCCACT | NM01106 | 335 |
| P2X5 | + | + | TGACCTTGATAAAGCTGCCTCCCA  TGCTATTCTGCTTCCTGCCACTCT | NM033321 | 482 |
| P2X6 | + | + | AGGGTACAACTTCAGGACAGCCAA  TCCACATACAGTAGCAGCAGGTCA | DY543046 | 213 |
| P2X7 | + | + | AACCCACAGCGCAAAGGAATCCAGA  AAGTCCAATGCACACAGTGGCCAAG | NM011027 | 637 |

b.

| Receptor | Cortical | Striatal | Primer 5’🡪3’ | Accession No | BP |
| --- | --- | --- | --- | --- | --- |
| P2Y1 | + | + | ACCTGCTATGACACCACGCCAAT  TCCAACCGTGCTCGCAAATTCATC | NM008772 | 735 |
| P2Y2 | + | + | CTGTGGTGCAAGCAATTCCACTGT  AGGCCAGTTTCAGCTACATGGCTA | NM008773 | 203 |
| P2Y4 | - | + | AGGCAGGCGTGAAGTGAG  AGAGGCAACAGGATGAACTTG | NM020621 | 207 |
| P2Y6 | + | + | CCGTGAGTTCTGCGTGTGTG  TCACGGTAGACGCAGGTGGT | NM183168 | 464 |
| P2Y12 | + | + | GCAACAGATGCCAGTCTGCAAGTT  AAATGGCCTGGTGGTCTTCAGGTA | NM027571 | 490 |
| P2Y13 | - | + | AAACAGAGCACCAGAAGAGAGGCA  GGATTTCACATTACAGGCGCGGTT | NM028808 | 236 |
| P2Y14 | + | + | GGTGAATGTGTTTGTGTGCAGGGT  TGACACCCTGGTTTGTCAGGATGA | NM133200 | 230 |

c.

| Receptor | Striatal | Primer 5’🡪3’ | Accession | BP |
| --- | --- | --- | --- | --- |
| TRPC1 | + | GATGTGCTTGGGAGAAATGCT  ACTGACAACCGTAGTCCAAAAG | NM011643 | 88 |
| TRPC2 | + | GCCATGTGGTGTCATTTTCCT  GTTGTCCAGTCTTGTCCTGAG | NM011644 | 114 |
| TRPC3 | + | GCCTTCATGTTCGGTGCTC  GGTCACCTCCAGATGCTCATT | NM019510 | 195 |
| TRPC4 | + | AAGGACCGCCCTCCTCATT  CGCATCGCCTACATAGACATTG | NM001253683 | 88 |
| TRPC5 | - | GGGCTGAGACTGAGCTGTC  TTGCGGATGGCGTAGAGTAAT | NM009428 | 247 |
| TRPC6 | - | AGCCAGGACTATTTGCTGATGG  AACCTTCTTCCCTTCTCACGA | NM013838 | 152 |
| TRPC7 | - | CTTCCTGGACTCGGCTGAGTA  GCGTTCTGCCCCATGTAGT | NM012035 | 105 |
